# Knowledge-based identification of sleep stages based on two forehead electroencephalogram channels

**DOI:** 10.3389/fnins.2014.00263

**Published:** 2014-09-04

**Authors:** Chih-Sheng Huang, Chun-Ling Lin, Li-Wei Ko, Shen-Yi Liu, Tung-Ping Su, Chin-Teng Lin

**Affiliations:** ^1^Brain Research Center, National Chiao-Tung UniversityHsinchu, Taiwan; ^2^Institute of Electrical Control Engineering, National Chiao-Tung UniversityHsinchu, Taiwan; ^3^Department of Electrical Engineering, Ming Chi University of TechnologyNew Taipei City, Taiwan; ^4^Institute of Bioinformatics and Systems Biology, National Chiao-Tung UniversityHsinchu, Taiwan; ^5^Department of Biological Science and Technology, National Chiao-Tung UniversityHsinchu, Taiwan; ^6^Department of Psychiatry, Taipei Veterans General HospitalTaipei, Taiwan

**Keywords:** sleep quality, sleep stages, polysomnography (PSG), electroencephalogram (EEG), sleep stage classification system

## Abstract

Sleep quality is important, especially given the considerable number of sleep-related pathologies. The distribution of sleep stages is a highly effective and objective way of quantifying sleep quality. As a standard multi-channel recording used in the study of sleep, polysomnography (PSG) is a widely used diagnostic scheme in sleep medicine. However, the standard process of sleep clinical test, including PSG recording and manual scoring, is complex, uncomfortable, and time-consuming. This process is difficult to implement when taking the whole PSG measurements at home for general healthcare purposes. This work presents a novel sleep stage classification system, based on features from the two forehead EEG channels FP1 and FP2. By recording EEG from forehead, where there is no hair, the proposed system can monitor physiological changes during sleep in a more practical way than previous systems. Through a headband or self-adhesive technology, the necessary sensors can be applied easily by users at home. Analysis results demonstrate that classification performance of the proposed system overcomes the individual differences between different participants in terms of automatically classifying sleep stages. Additionally, the proposed sleep stage classification system can identify kernel sleep features extracted from forehead EEG, which are closely related with sleep clinician's expert knowledge. Moreover, forehead EEG features are classified into five sleep stages by using the relevance vector machine. In a leave-one-subject-out cross validation analysis, we found our system to correctly classify five sleep stages at an average accuracy of 76.7 ± 4.0 (*SD*) % [average kappa 0.68 ± 0.06 (*SD*)]. Importantly, the proposed sleep stage classification system using forehead EEG features is a viable alternative for measuring EEG signals at home easily and conveniently to evaluate sleep quality reliably, ultimately improving public healthcare.

## Introduction

Monitoring human physiology during sleep is essential for individual health. Sleep is increasingly viewed as playing an important role in restitution (Akerstedt et al., 2007). As an important aspect of well-being, sleep quality is closely related to overall quality of life, life satisfaction, secretion of the stress hormone, cortisol, and inadequate immunity (Gallagher et al., 2010). Evaluating of sleep quality is especially relevant, owing to a considerable number of pathologies linked to the sleep. Sleep stages are also recorded for clinical diagnosis and the treatment of sleep disorders. Sleep quality is most closely related to the distribution of depth of sleep; indeed, sufficient sleep quality must reach adequate deep sleep. The depth of sleep is characterized by different cortical electrical activities. Several sleep stages can be defined by variations of cortical electrical activities and other physiological signals, i.e., muscle activity and eye movement. According to Rechtschaffen and Kales rules (R&K rules), sleep stage can be segmented into wakefulness, movement time (MT), REM and sleep stages S1, S2, S3, and S4 based on features of EEG, EOG, and EMG (Kales and Rechtschaffen, 1968). In addition to modifying the standard guidelines for sleep classification by R&K, the American Academy of Sleep Medicine (AASM) developed guidelines for terminology, recording method, and scoring rules for sleep-related phenomena (Iber et al., 2007). In the AASM guidelines, sleep stages S1 to S4 are referred to as NREM stage 1 (N1), NREM stage2 (N2), and NREM stage3 (N3). N3 reflects slow wave sleep (SWS, R&K stages S3 + S4).

As the reference standard clinical multi-parametric system, polysomnography (PSG) (Holland et al., 1974) is used in sleep studies to define the different physiological sleep stages and diagnose many sleep disorders, including narcolepsy, restless legs syndrome, rapid eye movement (REM) behavior disorder, parasomnias, and sleep apnea. The PSG system requires a minimum of 11 channels, including electroencephalogram (EEG), electromyogram (EMG), electrooculogram (EOG), oxygen saturation (SpO2), and electrocardiogram (ECG). However, assessing a complete PSG has several limitations. First, PSG is not a portable device and typically placed in a sleep center, which is unfamiliar environment for patients. Second, PSG requires many physiological electrodes and wires placed on the scalp and body, possibly affecting their sleep further. Third, a standard sleep diagnosis in clinical practice is time-consuming and expensive (Zoubek et al., 2007). These processes are monotonous, and time consuming and unproductive. A simpler EEG acquisition and analysis system must be operable by patients for home use, as well as solve current PSG problems.

Recent studies have adopted bioelectrical signals (i.e., EEG, ECG, EMG, and EOG signals), which allow subjects to operate at home in order to develop sleep stage scoring methods, while attempting to obtain results similar to those of experts involved in visual scoring (Park et al., 2000; Anderer et al., 2005; Tian and Liu, 2005; Berthomier et al., 2007; Doroshenkov et al., 2007; Virkkala et al., 2007; Wang et al., 2009; Güneş et al., 2010; Jo et al., 2010; Yιlmaz et al., 2010; Eiseman et al., 2011). The classification structure of most of sleep stage classifications consists of feature extraction and classification schemes. The references differ from each other not only in the presented feature extractions and the corresponding classification schemes, but also in different bioelectrical signals used, such as EEG, EOG, or ECG. Feature extraction is a highly efficient means of achieving a satisfactory classification performance in order to develop a sleep stage classification approach. Certainly, if extracted features can achieve a high separability in distinguishing between different classes, classifiers can perform satisfactorily. In recent studies (Berthomier et al., 2007; Doroshenkov et al., 2007; Güneş et al., 2010; Jo et al., 2010), the signal process procedure regards an entire 30-s epoch as a processing unit to extract spectral and temporal information directly. The specific characteristics of sleep stages are smoothened easily within an entire 30-s signal process. For instance, the k-complex and sleep spindle only appear suddenly in a short period with 0.5–1.5 s in a 30-s epoch. Therefore, a short-term signals process should be incorporated when a developing feature extraction approach. Compared with EEG measurement, despite overcoming the hair problem, EOG and ECG still have certain limitations. For instance, EOG and ECG requires adhesive electrode pads, and the locations of EOG (or ECG) and corresponding amplifier are divergent from each other. A subject's sleep position may interfere with the wire, thereby degrading the EOG and ECG signal quality. Despite the persistent hair and conductive gel problems associated with EEG measurement, recent developments to resolve these problems include a headband and portable EEG recording device, as well as a dry polymer foam electrode for long-term EEG measurement (Lin et al., 2008, 2011). Additionally, physiological characteristics during sleep are more easily identified by EEG than by EOG and ECG, explaining why the former is preferred when classifying sleep stages.

Berthomier et al. (2007) assessed an automatic sleep scoring software (ASSEEGA). The system adopts a 3-step procedure for automatic sleep scoring, based on a single EEG channel. In classifying five sleep stages, the agreements between ASSEEGA and two expert manual scorings are 76.0% (kappa = 0.67) and 78.2% (kappa = 0.69). Although highly promising for diagnostic and automatic ambulant scoring. This system still requires 2 bipolar channels (Cz-Pz, international 10–20 standard system), which are located at the back of the skull and hair site, conductive gel, and a laboratory EEG recording device to achieve a high resolution. The connection between hair and conductive gel, and subject's sleep position may also worsen the EEG quality, further lowering the estimation accuracy of the sleep stage.

This work develops a sleep stage classification system via two forehead EEGs, i.e., FP1 and FP2. FP1 and FP2 EEG measurements have the following advantages: non-hairy site EEG recording is performed; the two signals also contain eye movement information; and the system is easily self-adhesive and self-applicable for homecare users and long-term monitoring. The proposed classification system incorporates a novel feature extraction approach, capable of extracting spectral information while considering manual scoring rules. The proposed system further incorporates the relevance vector machine (RVM) as the basic classifier. Importantly, the proposed system provides preliminary results for diagnostic assistance and automatic ambulant scoring to determine whether a patient requires detailed testing with the PSG system in a sleep laboratory. Furthermore, the headband and portable EEG device as well as dry EEG electrodes (Lin et al., 2008, 2011) greatly facilitate the implementation of the proposed system in homecare setting for long-term monitoring of sleep quality, as well as for large-scale population studies.

## Materials and methods

### Subjects and data acquisition

Ten right-handed adults participated in our study (ten males; mean age 24 ± 6 years). None of the participants reported having a history of psychological disorders. Following a detailed explanation of the experimental procedure, all participants completed a consent form before the experiment. To avoid influences from other external factors, all subjects were instructed not to consume alcoholic or caffeinated drinks or sleeping pills beforehand. The experiments were performed at night (10:00 p.m.–08:00 a.m.). All experimental procedures received approval from the local ethics committee (Institutional Review Board of Taipei Veterans General Hospital, Taiwan).

**Figure 1:**
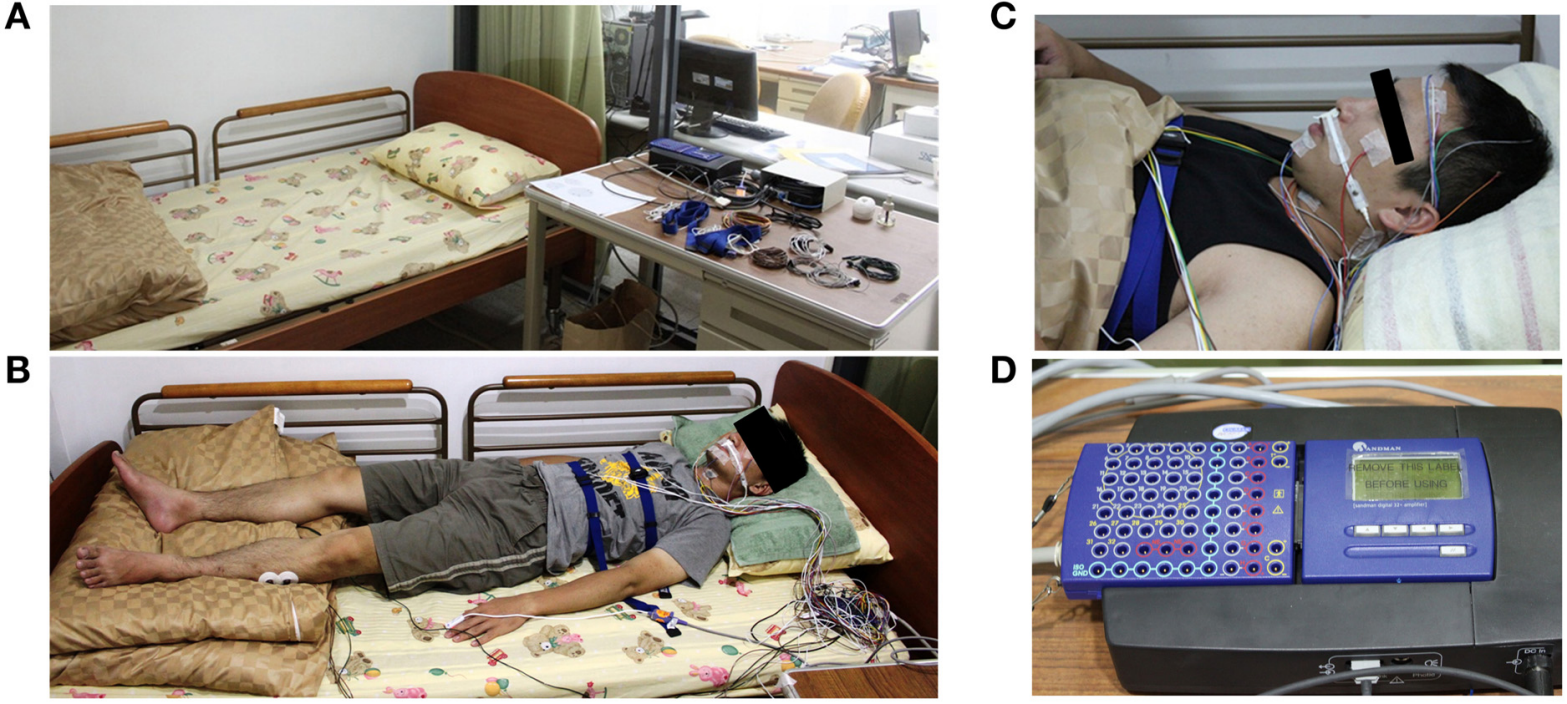
**Experiment environment and the polysomnography recording device (Holland et al., 1974). (A)** The experiment environment in the sleep laboratory of National Chiao-Tung University. **(B)** The real situation with complete PSG recording. **(C)** The FP1 and FP2 EEG channels are also recorded by the same PSG system simultaneously. **(D)** The PSG amplifier (Sandman Elite, Nellcor Puritan Bennett [Melville] Ltd., Kanata, Ontario, Canada).

The sleep PSG signals were recorded with a sampling rate of 128 Hz using Sandman Elite (Sandman Elite, Nellcor Puritan Bennett [Melville] Ltd., Kanata, Ontario, Canada) (Figure 1D). All subjects were required to sleep for a single night in the sleep laboratory of National Chiao-Tung University (Figure 1A) and wore all PSG electrodes during sleep. The complete PSG recording contains six channels EEG (F3, F4, C3, C4, O1, O2), two channels EOG, chin EMG, leg EMG, airflow signals, lead-II ECG, oximetry, nasal pressure, snoring sounding, and body position (Figure 1B). Forehead EEG signals from FP1 and FP2 were also recorded by the same PSG system simultaneously (Figure 1C). All of the EEG signals were re-referenced to the opposite lateral mastoids (A1 and A2). The contact impedance between all of the electrodes and scalp was controlled to be lower than 5 kΩ. No adjustment or artificial removal techniques were applied to the data. Each data set contained 5–8 h of forehead EEG signals and complete PSG signals.

### Sleep stage manual scoring

Sleep data for each subject were scored visually based on the manual scoring rules of AASM to five sleep stages by an experienced sleep expert. The five sleep stages are W, N1, N2, N3, and REM, and each 30 s sequential epochs was assigned to a sleep stage. Table 1 summarizes the distribution of sleep stage belonging to subjects.

**Table 1:** **Distribution of sleep stages for each subject**.

**SC**	**W**	**N1**	**N2**	**N3**	**REM**
S01	286	132	263	117	92
S02	138	46	356	69	81
S03	214	85	182	111	14
S04	88	120	356	156	154
S05	98	124	265	99	87
S06	102	122	267	145	99
S07	301	94	239	148	100
S08	117	105	364	136	189
S09	71	135	336	170	275
S10	103	121	491	132	156
Ratio	18.4%	13.1%	37.8%	15.6%	15.1%

The number in each lattice represents the number of corresponding sleep stages. SC refers to the subject code.

### Proposed automatic sleep stages classification system

**Figure 2:**
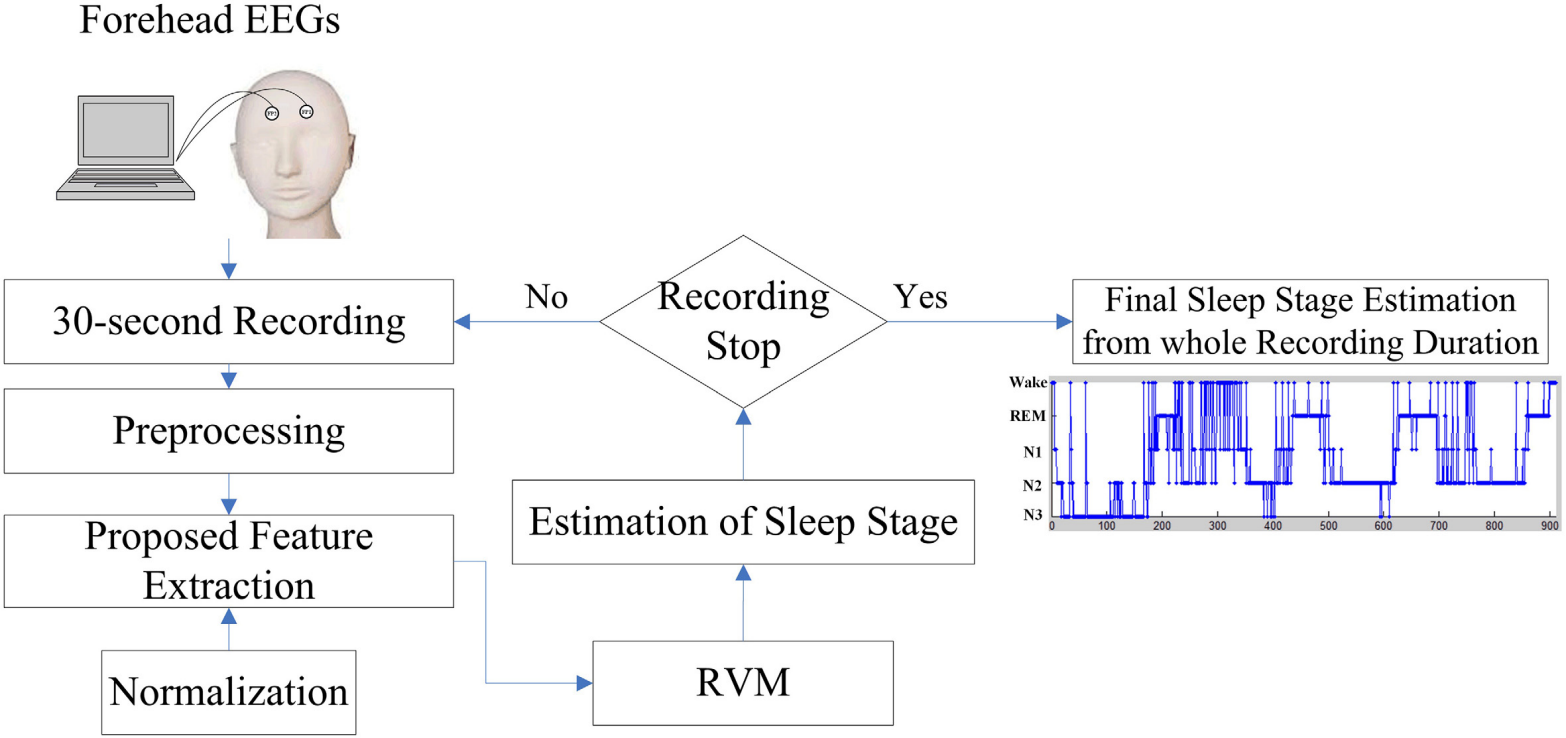
**Flowchart of the proposed sleep stage classification system based on the forehead EEG channels (FP1 and FP2)**.

Visual manual scoring often identifies the different sleep stages based on EEG activities. While attempting to incorporate the advantages of EEG activities of manual scoring rule, this work presents a novel sleep stage classification system embedded with a feature extraction approach, which is inspired by the sleep clinician's expert knowledge in translating two forehead EEG signals to the relevant features, and relevance vector machine (RVM) in order to classify the sleep stages automatically. Figure 2 displays the flowchart of the proposed sleep stage classification system. As per AASM recommendations, a 30-s sequential EEG recording should function as a unit to assign a sleep stage. In the preprocessing step, all of the 30-s EEG signals are filtered by a band pass filter within 0.5–50 Hz. The following sections described the proposed feature extraction, normalization, and RVM procedures in detail. After RVM, the input 30-s EEG recording assigns a sleep stage. When the recording procedure stops, the final sleep stage results for the whole recording can be estimated.

#### Feature extraction

Previous studies extracted frequency-domain features by fast Fourier transform (FFT) within the entire30-s signals. However, the previous studies regarded the entire 30-s signals as a processing unit to directly extract frequency-domain features by FFT. Under this circumstance, the specific characteristics of power spectrum density of 30 s signals are easily smoothened, and the corresponding sleep spectral activities are lost, when the characteristics of sleep appear only at a short period in the time signals. The entire 30 s signals contain a significant amount of information, and the spectral information obtained from FFT directly cannot accurately reflect the advantages of the manual scoring rules. To resolve this problem, this work presents a novel feature extraction approach to extract spectral features by short-time Fourier transform and manual scoring knowledge, which retain the properties of temporal manual scoring rules and represent the spectral response in power spectral density. According to the manual scoring rules of AASM (Iber et al., 2007), the EEG activities include alpha rhythm, theta rhythm, K complex, sleep spindle and slow waves. For instance, the epoch is scored the wakefulness when more than 50% of the epoch has alpha (8–12 Hz) rhythm. The epoch is scored as the N1 when alpha rhythm is attenuated and replaced by low amplitude, predominantly theta (5–7 Hz) rhythm for more than 50% of the epoch. The epoch is scored as N2 when the K complex or sleep spindle (12–14 Hz) occurs with the theta background rhythm. The epoch is scored as N3 when more than 20% of the epoch has high amplitude slow wave activity. If a single epoch contains 2 or more stages, the stage that contains the greatest portion of the epoch is assigned.

**Figure 3:**
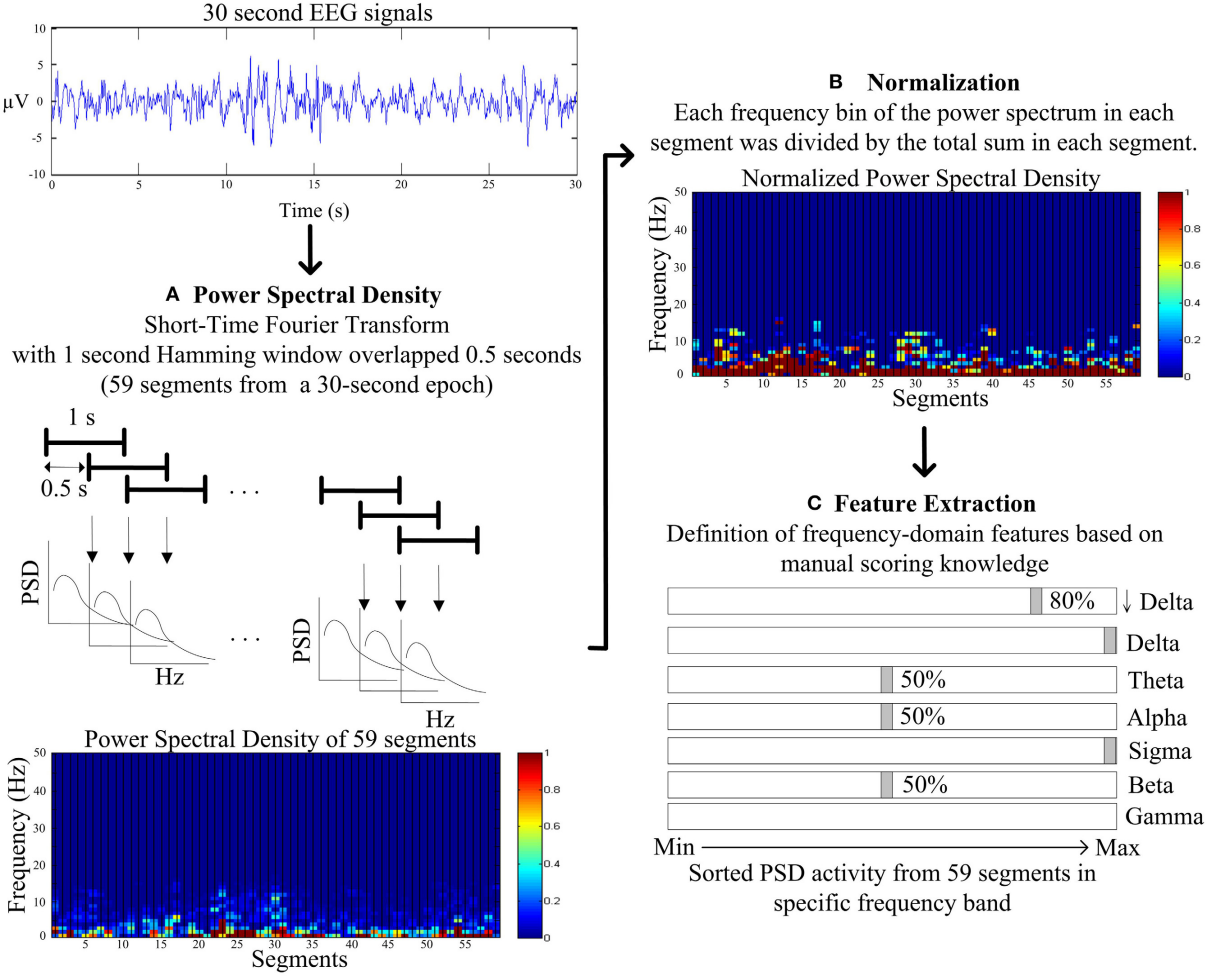
**Flowchart of the proposed feature extraction. (A)** The power spectral density (PSD) by the short-time Fourier transform (STFT) with a 1 s Hamming window overlapped with a 0.5 s window. **(B)** The normalization for PSD of each frequency bin of each segment is divided by the total PSD of each segment. **(C)** The frequency-domain feature extraction based on the manual scoring knowledge.

Figure 3 describes the proposed feature extraction for each 30-s EEG signal. Following the expert knowledge of manual scoring rules, the short-time Fourier transform (STFT) with a 1 s Hamming window overlapped with a 0.5 s window is used rather than using FFT within the entire 30-s signals (Figure 3A). Power spectrum densities (PSD) of fifty-nine segments are calculated after using STFT in entire 30-s EEG signals. Following STFT, the PSD of each segment is normalized to avoid individual differences. PSD of each frequency bin of each segment is divided by the total PSD of each segment (Figure 3B). Proposed features are identified in the following definition.

The slow wave activity (0.5–2 Hz and amplitude of greater than 75 microvolt) refers to an important index to score N3, and the ratio of slow wave activity has to be greater than 20% visually. Therefore, PSD in lower delta (0.5–2 Hz, denoted as ↓Delta, Figure 3C) is chosen to represent the feature of the slow wave activity. Moreover, the average PSDs of the lower delta of upper and lower 80% of 59 segments are viewed as the features of slow wave activity.

As the most important characteristics in visually identifying N2, and K complex and sleep spindle occur spontaneously and roughly every two epochs. K complex is a well-delineated negative sharp wave in EEG immediately followed by a positive component with total duration at least 0.5 s; in addition, sleep spindles are oscillations of sigma (12–14 Hz) with duration of 0.5–1.5 s. Hence, the maximum PSD of sigma band among 59 segments and the average PSD of sigma band of the remaining 58 segments is represented as features of sleep spindle. Moreover, the maximum PSD of delta band (1–4 Hz) among 59 segments and the average PSD of delta band of the remaining 58 segments are represented as features of K complex (Figure 3C).

While tending to appear during drowsy, meditative, and sleep onset, theta rhythm scores the epoch as N1, N2, and REM. The average PSDs of theta of upper and lower 50% of 59 segments represent the features of light sleep (N1+N2) (Figure 3C).

Movement time, normal resting waking consciousness and wakeful relaxation with eyes closed are accompanied by gamma rhythm (30–50 Hz), beta rhythm (15–30 Hz) and alpha rhythm, respectively. Experts score the epoch as stage wakefulness, when beta and alpha rhythm appear more than 50% of epoch. Thus, the average PSDs of beta and alpha band of upper and lower 50% of 59 segments are represented as the features of stage wakefulness. Movement time stage is mainly accompanied by muscle artifacts obscuring the EEG for more half an epoch. Hence, the average PSD of gamma of 59 segments is represented as the feature of movement time stage (Figure 3C).

As mentioned earlier, for FP1 and FP2 EEG channels, sixteen features are extracted, respectively. Two features are extracted as ↓delta activity; two features are extracted as delta activity; two features are extracted as theta activity; two features are extracted as alpha activity; two features are extracted as sigma activity; two features are extracted as beta activity; and one feature is extracted as gamma activity.

For investigating the influence of the proposed feature extraction approach, the conventional PSD feature extraction approach is compared with the proposed one. The conventional PSD feature extraction approach is calculated by the fast Fourier transformation directly for each entire 30-s EEG signal. Notably, this work does not further consider the feasibility of integrating the frequency bins to the specific frequency bands such as delta and theta. The PSD activity ranging from 1 to 50 Hz is used here as input features. Also, for FP1 and FP2 EEG channel, fifty features are extracted, respectively.

#### Relevance vector machine

Relevance vector machine (RVM) is a learning algorithm based on Bayesian framework and support vector machine (SVM) (Tipping, 2001). RVM has a form similar to that of SVM; they differ in the measurement between binary classes. SVM learns the maximal distance of margins between binary classes, while RVM learns the maximal probability of margins between binary classes. In contrast with RVM, SVM has the following disadvantages: the number of support vectors (SVs) grows with an increasing number of training patterns; the overfitting problem may occur if SVM selects too many SVs; the decision value is derived from the hyperplane function of SVM in the feature space, making its formation as the probability degree impossible; and the penalty parameter of SVM must be set; this penalty parameter significantly influences the classification results. This parameter is generally determined by the cross-validation approach. Further details of RVM and SVM can be found in Tipping (2001).

### System performance validation

To illustrate the efficiency of the proposed feature extraction approach, this work evaluates the separability of different feature extraction approaches by using the Fisher criteria (Fukunaga, 1990). Two Fisher criteria are expressed as follow:

$$\begin{array}{l} J_{1}=\frac{tr(S_{b})}{tr(S_{w})}\\ J_{2}=tr(S_{w}^{-1}S_{b}) \end{array}$$

where ***S_b_*** and ***S_w_*** denote the between-class and within-class scatter matrix, and ***tr (A)*** refers to the trace of square matrix ***A***. A larger ***J*_1_** and ***J*_2_** imply a larger separability of the presented features in feature space.

Under the extracted feature approaches, this work compares the classification performances of linear discriminate analysis (LDA) (Fukunaga, 1990), k-nearest neighbor classifier (k-NN) (Fukunaga, 1990), SVM (Chang and Lin, 2011; Li et al., 2012), and RVM (Tipping, 2001). Several trials are performed for k-NN, in which the value of k is varied from 1 to 20, to determine the value that maximizes the accuracy. The selected k in k-NN is 13. For simplicity, this work only adopts the linear kernel for SVM and RVM to evaluate how the proposed feature approach influences. From the perspective of SVM and RVM, the advantage of SVM and RVM is the extension of feature space by the kernel function. The feature space of SVM and RVM is implicitly defined by the kernel function. Hence, two popular kernel functions, i.e., linear and radial basis function (RBF) kernel of SVM and RVM, are more closely examined. As for the SVM, a penalty parameter (also called slack variable) C of SVM, in which the trade-off between the margin and the size of the slack variables in this experiment is controlled, is determined by a grid search within given set {0.1, 0.5, 1, 10, 50, 100, 500, 1000, 1500}. Here, the C selected from the grid search is 50. A grid search is also performed to derive the proper parameter of RBF kernel within a set {0.1, 0.25, 0.5, 0.75, 1, 2, 5, 10, 50, 100, 1000}. Here, the selected parameter of RBF for both SVM and RVM is 0.5.The multiclass strategy in SVM and RVM adopted in this work is a one-against-all strategy (Bottou et al., 1994; Li et al., 2012).

In this work, the sleep PSG data of ten subjects are collected. To evaluate the performance of the proposed classification system, the classification performance is evaluated using leave-one-subject-out cross validation (LOSO). Implementing LOSO involves taking the data from one subject as the testing set and the data from other remaining subjects as the training set; the same procedure is repeated until all subjects are including in the testing set. As is well known, the training data and the testing data should be independent of each other. Restated, the testing information should not be used in the training step. The k-fold cross validation approach is the conventional means of evaluating the classification performance. However, this approach cannot ensure that the training data and testing data are independent. Because the training data and testing data are from different subjects, the training data and the testing data in LOSO are independent. Hence, LOSO is less subjective than the normally adopted k-fold cross validation within the single subject.

This system performance is evaluated using three valid indices, i.e., overall accuracy, sensitivity, and Cohen's kappa coefficient. Overall accuracy refers to proximity of measurement results to the actual value and precision to the repeatability or reproducibility of the measurement. Sensitivity is performed to reflect the ability to identify positive results for each class. Cohen's kappa is a statistical measure of inter-rater agreement or inter-annotator agreement for qualitative (categorical) items (Cohen, 1960). As is generally assumed this measurement is more robust than simple percent agreement calculation since kappa takes into account the agreement occurring coincidentally.

## Experimental results

**Figure 4:**
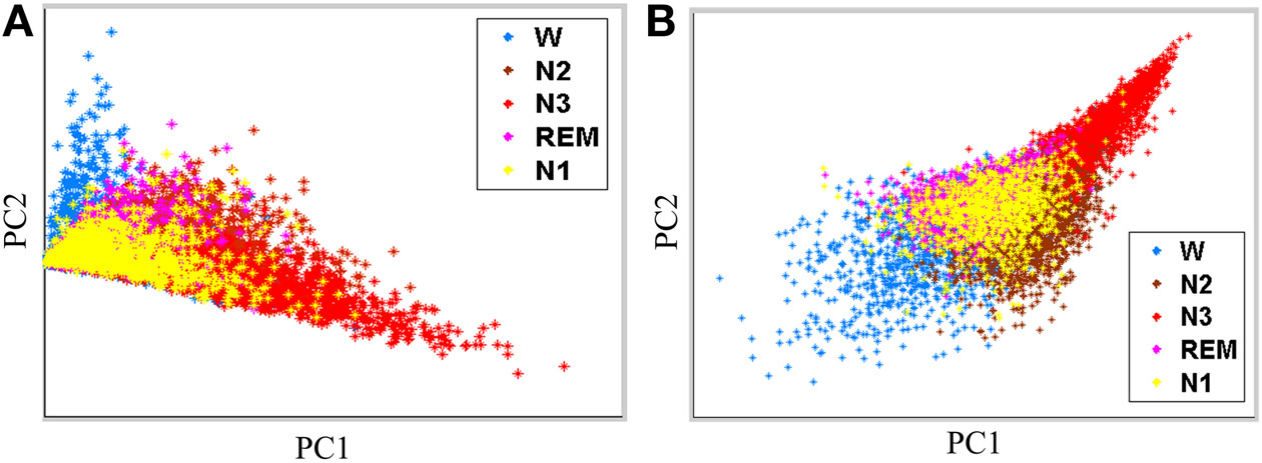
**Feature scatter plots with first two principal components (PCs) by (A) the conventional PSD feature extraction, and (B) the proposed feature extraction**.

Performance of the proposed feature extraction approach is evaluated by using Fisher criteria, i.e., ***J*_1_** and ***J*_2_**, to demonstrate the separability. Table 2 lists the values of ***J*_1_** and ***J*_2_**. Both of ***J*_1_** and ***J*_2_** in the proposed feature extraction approach are larger than the conventional PSD feature extraction approach, implying that the proposed feature extraction approach has a better separability than conventional PSD feature extraction approach. For illustration, principal component analysis (PCA, Fukunaga, 1990) is performed to decompose the proposed extracted features and conventional frequency PSD features to first two principal components (PCs), respectively. Figures 4A,B show the scatter plot of the first two PCs from the conventional PSD feature extraction approach and the proposed feature extraction approach, respectively. These figures clearly reveal that the spatial distribution of the proposed feature extraction in PC space has the better scatter distribution than that of the conventional PSD feature extraction one. The scatter plot of the proposed feature extraction approach presents the scatter points from different groups, i.e., wakefulness, N2, N3, and REM, which are leading ones in their own industries. However, the conventional PSD feature extraction approach can not verify this observation. Most data points from the conventional PSD feature extraction approach are mixing in PC space. Additionally, regardless of in which feature extraction approaches, most of the data points of N1overlap with N2, and REM, because the EEG characteristics of N1 in manual scoring rules closely resemble that of N2, REM.

**Table 2:** **Separability measurements by using Fisher criterion for the conventional PSD feature extraction and the proposed feature extraction approach**.

	**Conventional PSD feature extraction**	**Proposed feature extraction**
*J*_1_	0.69	0.74
*J*_2_	1.82	4.57

**Figure 5:**
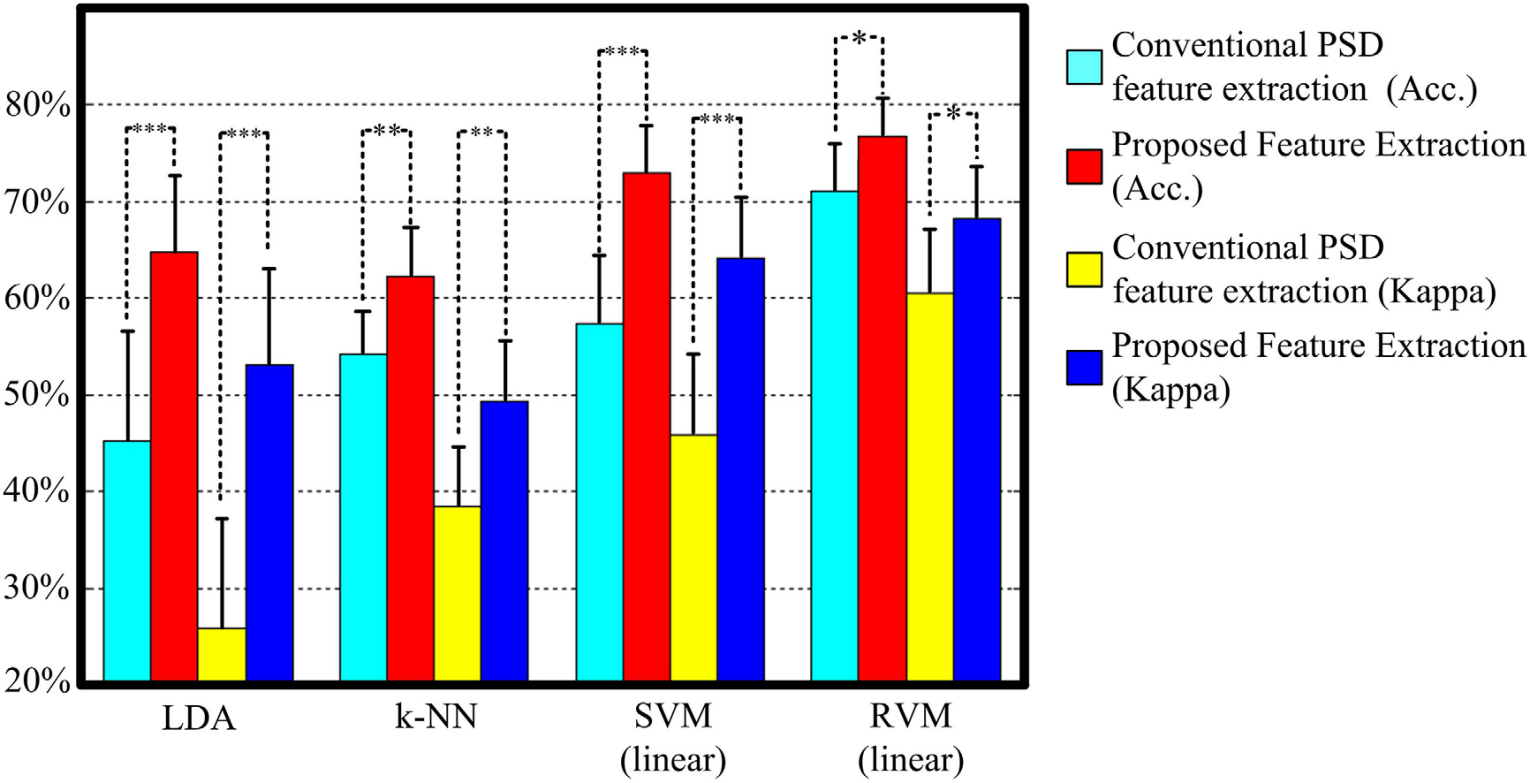
**The classification performance comparison between conventional frequency PSD feature extraction and the proposed feature extraction with LDA, k-NN, SVM, and RVM**. Acc. and kappa represents overall accuracy and Cohen's kappa coefficient, respectively. Error bars indicate standard deviations. For better visualization, the kappa values have been scaled with the factor 100. ^*^p < 0.05, ^**^p < 0.01, ^***^p < 0.001.

Figure 5 and Table 3 display the average classification performances from two feature extraction approaches and four classifiers. The performances, both in terms of overall accuracy and kappa coefficient, of the proposed feature extraction more significantly improves (*p* < 0.05, paired *t*-test) than the conventional frequency PSD features. The proposed feature extraction approach has an approximately 20% greater increase in overall accuracy and kappa coefficient than the conventional frequency PSD extraction approach. The overall accuracy and kappa coefficient can reach as high as 76.7 and 68.2% by RVM, respectively.

**Table 3:**
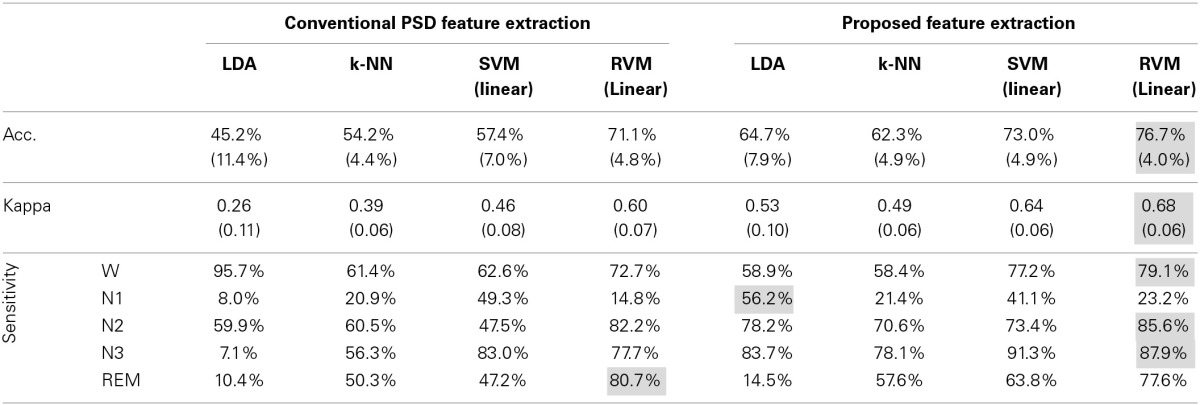
**Comparison of feature extraction approaches in terms of classification performance**.

Each lattice represents the mean value of the validation index from ten subjects, and the corresponding bracket is the standard deviation. Acc. and kappa represents overall accuracy and Cohen's kappa coefficient, respectively. Highlighted parts display the optimum performance of all comparison results.

Figure 4 shows scatter plots of the sleep pattern, while Table 2 lists the separability values of Fisher criteria, which compare the features of the conventional extracted PSD with those of the proposed approach. According to the above results and Fisher criteria, the proposed feature extraction approach has a better separability than the conventional PSD feature extraction approach, as also verified by the classification performance in Table 3. The rise in classification performance depends on the proposed feature extraction approach while considering the manual scoring criteria. In particular, the kappa coefficient in the proposed feature extraction approach is increasing significant implying that the proposed method improves both the overall performance of classification and its accuracy for each class with a balance trade-off. For instance, the sensitivity for wakefulness in LDA (conventional PSD feature extraction approach) is 95.7%, i.e., the highest sensitivity of a single class; however, the sensitivity of the other classes is extremely low.

**Figure 6:**
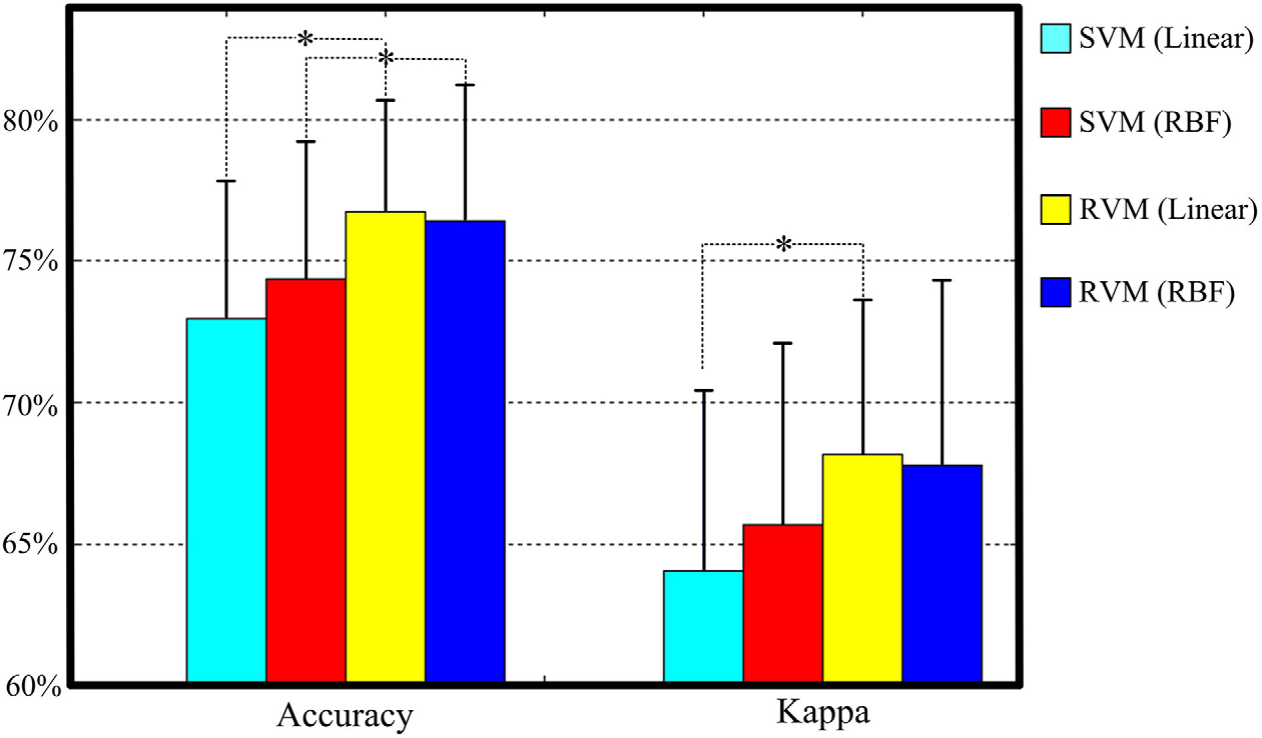
**The classification performance comparison between SVM and RVM with linear and RBF kernel functions**. Acc. and kappa represents overall accuracy and Cohen's kappa coefficient, respectively. Error bars indicate standard deviations. For better visualization, the kappa values have been scaled with the factor 100. ^*^p < 0.05.

Table 4 and Figure 6 summarize the results of SVM and RVM with RBF kernel and liner kernel. The optimum result is RVM with linear kernel, in which the accuracy and kappa can reach 76.7% and 0.68, respectively. Next, RVM and SVM are compared, revealing a significant increase in both linear kernel function (over accuracy, *p* = 0.012, paired *t*-test; kappa coefficient, *p* = 0.024, paired *t*-test) and RBF kernel function (over accuracy, *p* = 0.033, paired *t*-test; kappa coefficient, *p* = 0.069, paired *t*-test). Two kernel functions in SVM and RVM are also compared. Applying the RBF kernel in SVM has a ~1.5% improvement in overall accuracy and kappa coefficient. However, it does not reach a statistically significant level (over accuracy, *p* = 0.071, paired *t*-test; kappa coefficient, *p* = 0.089, paired *t*-test). In terms of RVM, applying the RBF kernel in RVM also does not reach a statistically significant level (over accuracy, *p* = 0.582, paired *t*-test; kappa coefficient, *p* = 0.658, paired *t*-test).

**Table 4:**
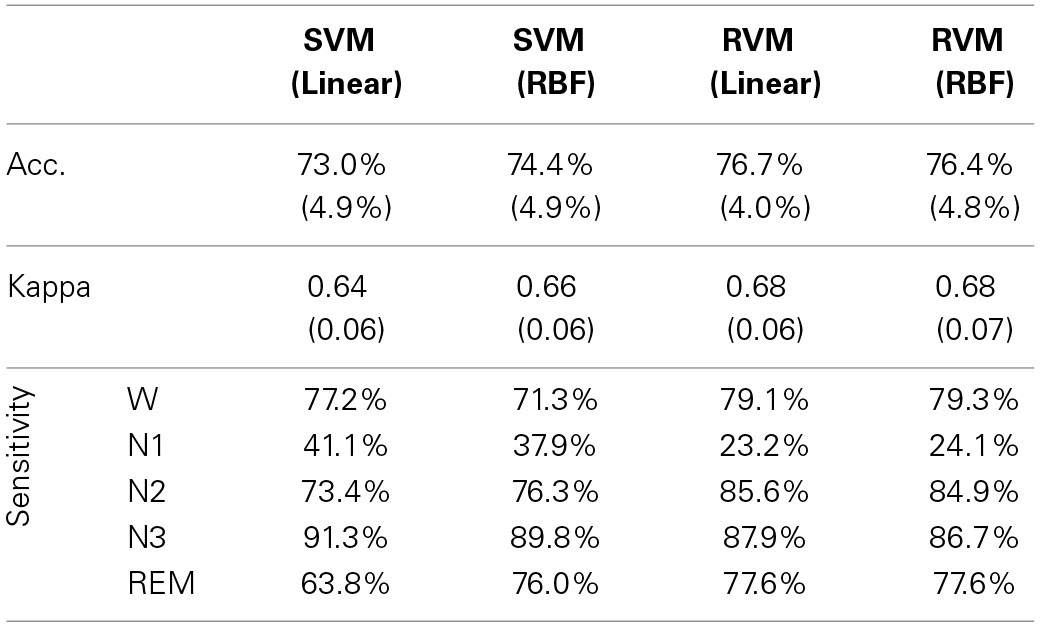
**Performance comparison of different kernel functions in SVM and RVM**.

Each lattice represents the mean value of the validation index from ten subjects, and the corresponding bracket is the standard deviation. Acc. and kappa represents the overall accuracy and Cohen's kappa coefficient, respectively.

In terms of SVM, although the number of SVs is normally less than the training patterns, the number of SVs grows larger with an increasing number of training patterns. However, the overfitting problem occasionally occurs in SVM with a large number of SVs. The number of SVs of SVM is generally larger than that of RVs in RVM. The number of support vectors (SVs) is also compared with that of relevance vectors (RVs). RVM is characterized by the fewer number of RVs than that of SVs in SVM.

Fewer RVs can avert the overfitting problem. For each subject, the training patterns come from the remained nine subjects, explaining the variation in the number of training patterns. This work adopts the one-against-all multiclass strategy, which is a “one class vs. all others” method, for SVM and RVM. Therefore, the train population is considered from all classes. In the one-against-all multiclass strategy, SVM and RVM train individual decision hyperplane (formula of SVs or RVs and corresponding coefficients) for each class. From ten subjects, the mean of number of train pattern is 7425.9, and standard deviation is 131.4. Table 5 shows the mean of number of SVs and RVs from linear kernel and RBF kernel. Regardless of whether linear kernel or RBF kernel is adopted, the number of RVs is significantly less than the number of SVs (all *p*-values are less than 0.0001 with Student's *t*-test). Although the number of trained SVs is in the thousands, the number of trained RVs only ranges less than several hundred, thus representing a significant difference between RVs and SVs.

**Table 5:** **Number of support vectors in SVM and number of relevance vectors in RVM**.

	**W vs. others**	**N1 vs. others**	**N2 vs. others**	**N3 vs. others**	**REM vs. others**
SVM	1503.0	5276.9	3096.8	921.9	1988.4
(Linear)	(105.5)	(150.9)	(86.8)	(46.9)	(46.3)
RVM	18.7	15.9	20	16.5	22.4
(Linear)	(1.1)	(1.3)	(1)	(2.1)	(1.5)
SVM	7425.9	7425.9	7425.9	7425.9	7425.9
(RBF)	(131.4)	(131.4)	(131.4)	(131.4)	(131.4)
RVM	19.7	22.9	37.1	11.3	41.5
(RBF)	(1.4)	(6.5)	(2.7)	(2.0)	(1.4)

Each block is the mean from all ten subjects, and the corresponding bracket is the standard deviation.

To investigating how class imbalance prior influences the classification performance, this work describes the confusion matrix between the proposed sleep classification approach, in which RVM is used with linear kernel function and expert manual scoring (Table 6). The overall accuracy and kappa coefficient, as computed from this confusion matrix, are 76.7% and 0.69, respectively. Table 6 and the performance in Table 4 demonstrate that the reported validation indices are not biased.

**Table 6:**
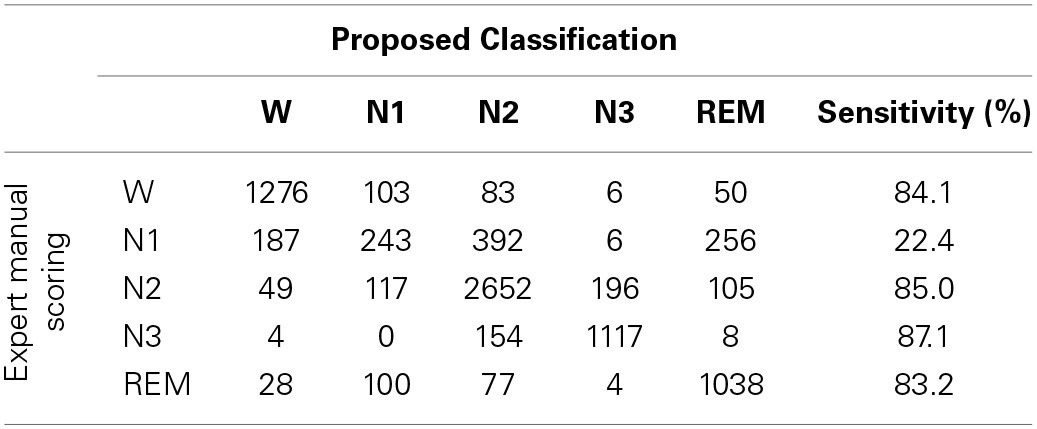
**Confusion matrix: proposed sleep classification approach vs. expert manual scoring**.

**Figure 7:**
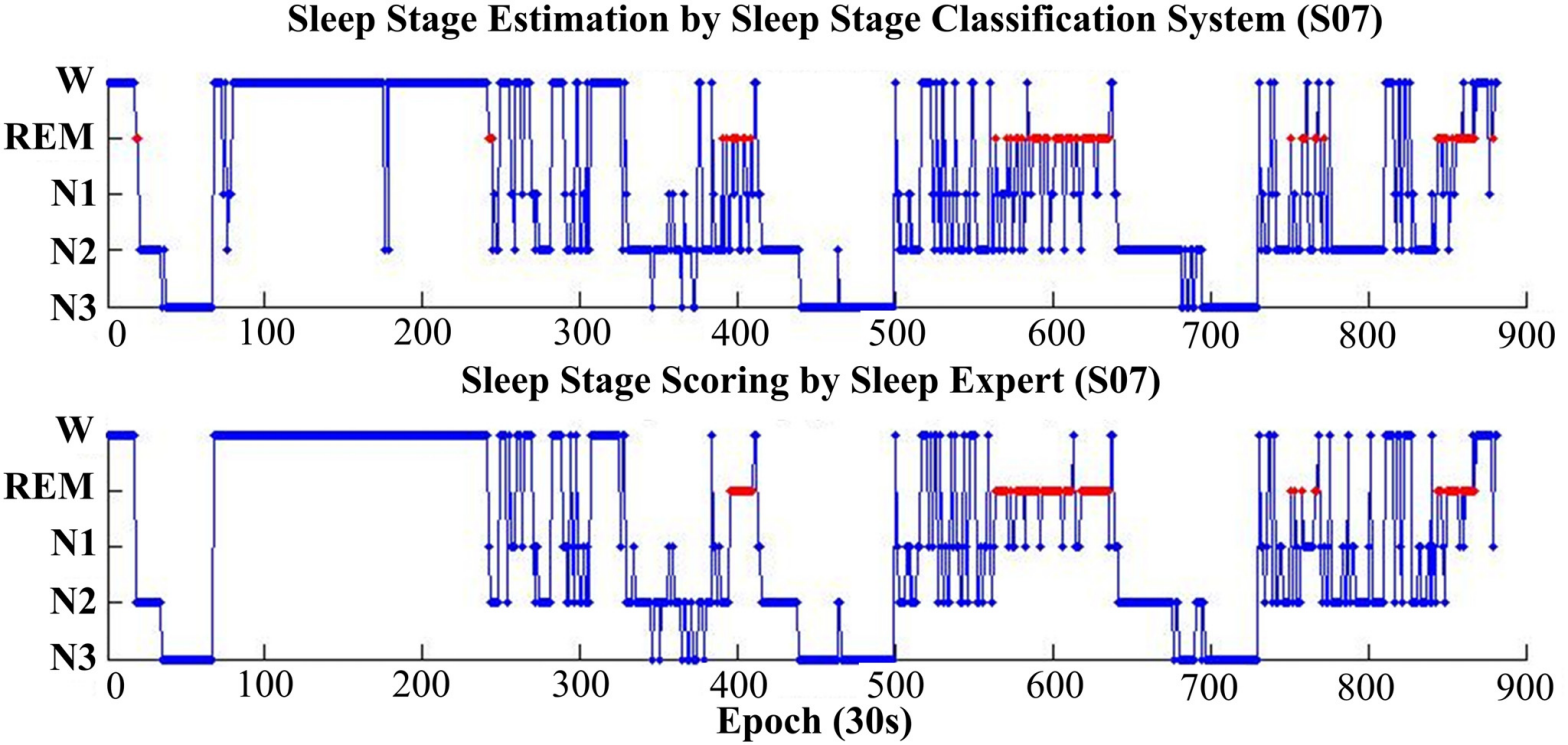
**Hypnograms obtained from the proposed sleep stage classification system (top plot) and the sleep expert with manual scoring rules (bottom plot)**. For this subject (S07), the overall accuracy is 82.5% (kappa 0.77).

Figure 7 shows the estimation results, based on the proposed classification approach and the manual scoring results for one subject (S07). The top plot is the distribution of the estimated sleep stages from proposed classification, and the below plot is the distribution of the sleep stages from the sleep expert. For this subject, the accuracy between the proposed classification approach and the sleep expert's scoring is 82.5% (kappa = 0.77). The proposed classification approach with only forehead EEG can reach a quite similar performance with the sleep expert.

## Discussion

Based on the scatter plot and Fisher criteria, this work demonstrates that the proposed feature extraction approach can achieve a better separability than the conventional PSD feature extraction approach. The classification performance also demonstrates that the proposed feature extraction approach is more effective than the conventional PSD feature extraction approach in distinguishing between different sleep stages. The proposed feature extraction approach is considered with the sleep stage manual scoring knowledge. Additionally, time-frequency analysis is performed to extract the spectral activities in a short segment window signal not whole 30-s signals. The proposed feature extraction approach characterizes not only by its temporal information of manual scoring, but also by its spectral response. Sleep experts distinguish between the different sleep stages by counting the rhythm and the amplitude of EEG visually by per-second EEG signals from whole 30-s signals. The proposed feature extraction approach applies the short-time Fourier transform with a one second window and overlap 0.5 s. Also, the PSD activity is transferred from STFT to several specific frequency bands, e.g., low delta (1–2 Hz), delta (1–4 Hz), theta (5–7 Hz), alpha (8–12 Hz), sigma (12–14 Hz), beta (15–30 Hz), and gamma (30–50 Hz). With the entire 30-s data, 59 segments are extracted. To overcome the individual differences, the PSD of each segment is divided by the total sum of the PSD of each segment. Hence, the PSD activity in the proposed feature extraction approach is the proportion response of PSD. Based on the expert's manual scoring knowledge, several features are extracted from these specific frequency bands of 59 segments. STFT can achieve the spectral activity of specific frequency response which may be more or less resonant in the spectral space. For instance, the sleep spindle is a sigma rhythm lasting from 0.5 to 1 s, and occurring suddenly. If the fast Fourier transfer is applied to a N2 epoch with one spindle of whole 30-s EEG signals, the specific sigma frequency response cannot be identified clearly. The principal spectral response might be a background spectral response, i.e., theta spectral response, and the amplitude of PSD within the sigma band might be extremely low. However, STFT can separate the signals into several segments in order to calculate the PSD individually. If the spindle occurs, STFT can enhance the corresponding spectral activity more than that of FFT with whole signals. Moreover, in addition, to using STFT to transfer the signals to PSD, this work also proposes sorted power activities to extract the manual rules' properties for the specific frequency spectral activity as the sleep features. The corresponding specific frequency spectral activities are extracted by following the manual scoring rules. For instance, the maximum power of sorted sigma power activities can represent the feature of the sleep spindle. If the current epoch is N2, the value of the maximum power of sorted sigma power activities is higher than that of the maximum power of sorted sigma power activities from the other sleep stages.

The value extracted by average PSDs of alpha and beta of upper 50% of 59 segments from wake stage is higher than that of sleep. The value extracted by the average PSDs of the slow wave of upper 80% of 59 segments from N3 stage is higher than that of other stages. Similarly, the other features from different frequency bands can represent the other sleep stages. Berthomier et al. (2007) characterized several contrast functions, which are feature extraction approaches, as defined by the EEG power activity calculating from whole epoch directly. Although Berthomier et al. (2007) considered the baseline resting EEG frequency of each individual to adjust the spectral criteria, the extracted features were still calculated from the whole 30-s EEG signals. The specific frequency activity still diminishes, when the frequency activity is calculated within the whole 30-s signals. Hence, the proposed feature extraction approach is a more effective means of extracting the sleep characteristics.

SVM has recently achieved higher empirical accuracy and better generalization capabilities than other standard supervised classifiers (Fatma Guler and Ubeyli, 2007; Lotte et al., 2007; Xu et al., 2009; Li et al., 2012). However, as mentioned earlier, SVM is limited in the number of SVs and the selection of penalty parameter. The penalty parameter in SVM is adjusts the generalization capability. RVM is an extension algorithm that eliminates the disadvantages of SVM. SVM learns the maximal distance of margins between binary classes while, in contrast, RVM learns the maximal probability of margins by exploiting a probabilistic Bayesian learning framework between binary classes. The penalty parameter in SVM is usually determined by the cross-validation approach. The chosen penalty parameter depends on the setting candidate set. Too much training time is expended when selecting the penalty parameter in SVM, if the setting candidate set has a wide range. The chosen penalty parameter is a local optimum parameter, depending on the setting candidate set, not the global optimum parameter. The chosen penalty parameter, which affects the number of SVs, incurs the overfitting problem in training phase. RVM can estimate penalty parameters automatically. Additionally, RVM can improve the problem in the number of SVs. Accounting to our results, the number of SVs can significantly decrease in the number of RVs; the classification performance can also be improved by RVM. Hence, the RVM is applied as the basic classifier in the proposed sleep stage classification system.

The detection of N1 is always the most problematic aspect of the sleep stages (Virkkala et al., 2007). Identifying a significant feature in EEG that could separate N1 from wakefulness, N2, and REM, is rather difficult because N1 is a transition phase in the changes of wakefulness and other sleep stages (Virkkala et al., 2007). The sleep EEG characteristics of N1 closely resemble those of N2, REM, and resting wakefulness. Our results demonstrate that the extracted features from PSDs in N1 resemble to N2, REM and resting wakefulness (Figure 4). Moreover, many epochs of N1 are misclassified to Wake, N2, and REM (Table 6), explaining the difficulty in automatically identifying N1 by a computer. Efforts are underway in our laboratory to address this problem.

Recent efforts have attempted to develop a more reliable sleep system with few bioelectrical channels, i.e., one EEG, one ECG, or two EOGs, in order to simplify the complex PSG inspection (Berthomier et al., 2007; Virkkala et al., 2007; Yιlmaz et al., 2010). Virkkala et al. (2007) devised an automatic sleep stage classification via two EOGs. The performances (Virkkala et al., 2007) with 5 sleep stages are 72.5% epoch-to-epoch agreement and 0.63 Cohen's kappa, and the sensitivity of Wake, REM, N1, N2, and SWS are 74.10, 72.7, 39.2, 79.1, and 73%, respectively. Although this work applies two channel signals, the FP1 and FP2 EEGs, which can reflect the eye movement, have more information in classifying sleep stages. Hence, both the characteristics of sleep EEG and eye movement are captured. Additionally, the proposed classification system accurately estimates sleep stages. Yιlmaz et al. (2010) presented a sleep stage and obstructive apneic epoch classification via single-lead ECG. The performances (Yιlmaz et al., 2010) with 6 sleep stages are 73.1% epoch-to-epoch agreement, and the sensitivities of Wake, REM, NREM1, NREM 2, NREM 3, and NREM 4 are 95.6, 84.9, 98.5, 61.8, 94.3, and 87.4%, respectively.

Performance of the classification (Yιlmaz et al., 2010) is satisfactory, even the sensitivity of NREM 1 (98.5%), which is the most difficult sleep stage to be identified automatically (Berthomier et al., 2007; Virkkala et al., 2007). Yιlmaz et al. (2010) applied the 10-fold cross validation within a single subject data, which totally separates a subject's self-data as the training data and also as the testing data. For instance, for a subject with total 800 epochs, partitioning produces 10 subsets with 80 epochs each. Therefore, the training set (720 epochs) and testing set (80 epochs) include totally separate sets of data. The training set and testing set originate from a specific subject. Moreover, the properties of training and testing data resemble each other, the corresponding with the over-fitting problem in training phase. Notably, attempting to use a subject-dependence model by a specific subject in order to classify another independent dataset may cause worst results. LOSO cross-validation is a more objective evaluation approach for machine learning experiment involving human subjects to allow for subject-to-subject variation. The testing data are subject-independent to the training data. Hence, the performance evaluation by LOSO is more effective and reliable than k-fold cross validation in developing a general model involving human subjects. As mentioned earlier, the right and left EOG signals are recorded by placing two electrodes at the nasal and temporal canthal regions of the eye, in which one electrode is attached to the middle of the forehead as ground electrode while another electrode is placed on the left mastoid M1 as reference electrode. ECG signals are acquired by two electrodes in a modified leads II configuration (Malmivuo and Plonsey, 1995). The positive and negative leads are placed on the fourth inter costal space and the left of the sternum. Also, both of the EOGs and ECG still require adhesive electrode pads, and the locations of EOGs (or ECG) and corresponding amplifier diverge from each other. The sleep position may affect the recording quality of physiological signals. Berthomier et al. (2007) presented an ASSEEGA based on an EEG channel. In terms of performance, the proposed sleep stage classification system is nearly equivalent to the ASSEEGA. However, ASSEEGA still requires 2 bipolar channels (Cz-Pz, international 10–20 standard system), making it infeasible for homecare applications. First, the position of electrodes is not easily identified, self-adhesive, and self-applicable for a self-operating user. Second, the conductive gel and a laboratory EEG recording device are still required in the recording signals. Although several portable EEG devices (e.g., Mindo-4S (Mindo, Hsinchu, Taiwan), MindWave Mobile (NeuroSky, CA, USA), and Emotiv epoc headset (Emotiv, Eveleigh NSW, Australia), as well as dry sensors) can overcome this problem, the comfort of dry electrodes of Cz and Pz is still a major challenge during sleep.

Anderer et al. (2005) recently developed and optimized an automatic classification system based on a central EEG channel, two EOG channels and a chin EMG channel; in addition, the final validation of overall epoch-by-epoch agreement is 80% (Cohen's kappa is 0.72) between the proposed automatic classification system and human expert scoring. Obviously, the data-rich recordings have more information, e.g., sleep brain electrical activity from EEG, muscle activity from EMG, and eye movement from EOGs. Such information-rich physiological data provide more important indices to classify the difference between REM and light sleep, i.e., the rapid eye movement and the lowest mandible muscle activity. Hence, the data-rich recording can achieve an excellent performance. Although the performance in this work is not equivalent to that of the classification system (Anderer et al., 2005), the proposed system attempts to reduce the number of full PSG signals to fewer physiological channels, as well as further classify the sleep stages effectively. Therefore, the proposed system uses forehead EEGs, i.e., FP1 and FP2. FP1 and FP2 have the following advantages: the physiological data include information from sleep brain electrical activity and eye movement; and adopting the forehead EEGs makes it feasible for self-application for a self-operating user. With the portable EEG device (Lin et al., 2008) and dry sensors (Lin et al., 2011), home-based users can easily to wear the EEG headband to record the sleep forehead EEG signals by self-applicable. Furthermore, we can further analyze with the collected data can be analyzed further, even leading to the development of on-line sleep stage classification software.

Despite its contributions, this work has certain limitations. The collected data are limited to young and healthy study participants. The sleep stages in normal person are expected to diverge from the norm and to be more heterogeneous than those of older or younger, healthy individuals or patients. The sleep stage manual scoring rules are based on counting the rhythm of different frequency activities. The proposed system attempts to comply with this criterion in order to extract the sleep features. The system also uses STFT as temporal and visual rules, i.e., processing the signal within one-second window, and further extracts features by PSDs representing different rhythms of different frequency activities. This system is created by the healthy subjects, and does not have obvious evidence to verify that the proposed sleep stage classification is reliable in older individuals or patients. Therefore, efforts are underway in our laboratory to study the relation between patients and the proposed sleep stage classification system.

## Conclusion

This work presents a novel sleep stage classification system, consisting of a novel feature extraction method and RVM classifier, based on only two forehead EEG channels. Also, the classification performance is consistent with the sleep clinician's expert knowledge. Experimental results demonstrate the feasibility of using the proposed system as the preliminary screening results for a preclinical diagnosis to assist clinicians in making a diagnosis (rather having a depth testing with PSG system in a sleep laboratory) to reduce time for the procedure. Moreover, the proposed system only uses two forehead EEG signals, allowing us to apply the wearable and wireless EEG recording device (Lin et al., 2008; Liao et al., 2012) [e.g., Mindo-4S (Mindo, Hsinchu, Taiwan) and MindWave Mobile (NeuroSky, CA, USA)] in order to record the patient's EEG signals at home. Importantly, the proposed system provides an easier way for large population studies, long-term sleep monitoring, and home-based daily care. Efforts are underway in our laboratory to integrate the wearable and wireless EEG recording device and the proposed sleep stage classification system. As an important aspect of performance, the automatic artifact detection might be a possible way to improve the efficacy of the proposed system. Hence, the efficacy of the automatic artifact detection should be considered in the proposed system in the future work.

### Conflict of interest statement

The authors declare that the research was conducted in the absence of any commercial or financial relationships that could be construed as a potential conflict of interest.
